# Exploring the association between 5 different alternative indicators of insulin resistance and the risk of multiple cardiovascular and metabolic diseases: A cross-sectional NHANES study from 2005 to 2018

**DOI:** 10.1097/MD.0000000000048080

**Published:** 2026-03-13

**Authors:** Xiuxia Song, Youfu He, Zhonggui Cai, Lei Peng

**Affiliations:** a General Family Medicine, Linping Hospital of Integrated Traditional Chinese and Western Medicine, Hangzhou, China; b Department of Cardiology, Guizhou Provincial People’s Hospital, Guiyang, China; c Department of Interventional Cardiology, Shandong Healthcare Group Zao Zhuang Hospital, Zaozhuang, China; d Department of Cardiology, Linping Hospital of Integrated Traditional Chinese and Western Medicine, Hangzhou, China.

**Keywords:** cardiometabolic multimorbidity, homeostasis model assessment of insulin resistance, insulin resistance, NHANES, predictive model

## Abstract

There is currently an absence of research exploring the correlation between insulin resistance (IR) surrogates and the risk of cardiometabolic multimorbidity (CMM). This study sheds light on the link between different IR surrogates to CMM risk and seeks to identify the optimal surrogate index for IR. Using the National Health and Nutrition Examination Survey 2005 to 2018 data, we applied logistic regression, the Boruta algorithm, trend tests, restricted cubic spline analysis, subgroup analysis, Brier scores, and receiver operating characteristic curve analysis to assess the relationship between CMM risk and IR markers including the triglyceride-glucose index (TyG index), the triglyceride-glucose-body mass index (TyG-BMI index), the metabolic score for insulin resistance (METS-IR), the triglyceride to high-density lipoprotein cholesterol ratio (TG/HDL-C ratio), and homeostasis model assessment of insulin resistance (HOMA-IR). The study included 15,537 participants, of whom 2881 developed CMM. Increased levels of IR markers were significantly associated with a higher CMM risk. Trend analysis showed a dose–response association (*P* for trend < .05). Restricted cubic spline analysis indicated a J-shaped nonlinear relationship for TyG index and ln[HOMA-IR] with CMM (*P* for overall < .001, *P* for nonlinear < .05). Conversely, ln[TyG-BMI], ln[METS-IR], and ln[TG/HDL-C] exhibited linear relationships with CMM (*P* for overall < .001, *P* for nonlinear > .05). HOMA-IR showed the highest area under the curve (0.699) for predicting CMM risk. As TyG, TyG-BMI, METS-IR, TG/HDL-C, and HOMA-IR levels increase, the CMM risk increases. Among these, HOMA-IR demonstrates a J-shaped nonlinear relationship with CMM risk and the best predictive performance.

## 1. Introduction

Cardiometabolic multimorbidity (CMM) represents a significant public health challenge in modern society, characterized by the coexistence of multiple metabolic and cardiovascular diseases like hypertension, diabetes, and coronary heart disease (CHD).^[1]^ Patients with CMM typically experience higher all-cause mortality and an increased incidence of cardiovascular events, significantly compromising quality of life and placing a significant financial strain on healthcare systems.^[2]^ Despite some progress in existing treatments for controlling a single disease, the intervention effect on complex comorbidities of CMM in the American population is still limited. In particular, there are no accurate risk screening tools, and thus it is difficult to identify high-risk groups early.^[3]^

Insulin resistance (IR) is a critical pathological mechanism underlying CMM,^[4]^ encompassing a range of pathological processes, including metabolic dysregulation, vascular dysfunction, and chronic inflammation.^[5]^ Studies have demonstrated that atherosclerosis, cardiovascular disease, and type 2 diabetes are all significantly influenced by IR.^[6]^ Nevertheless, conventional IR diagnosis methods like hyperinsulinemic–euglycemic clamp test, although highly accurate,^[7]^ are limited in their applicability to large population studies because they are expensive and intricate.^[8]^

In recent years, several IR surrogates based on clinical data have been proposed, including homeostasis model assessment of insulin resistance (HOMA-IR),^[9]^ the triglyceride-glucose index (TyG index),^[10,11]^ the triglyceride-glucose-body mass index (TyG-BMI index),^[12]^ metabolic score for insulin resistance (METS-IR),^[13]^ and the triglyceride to high-density lipoprotein cholesterol ratio (TG/HDL-C ratio).^[14]^ These indicators, by incorporating multiple metabolic parameters, not only reflect the associations between various metabolites and diseases but also offer the advantages of simplicity in calculation and cost-effectiveness. As a result, they have been widely used in clinical and epidemiological research in recent years, holding potential as tools for IR screening.^[15,16]^ However, current research on surrogate markers of IR remains limited in several respects. First, most existing studies primarily focus on predicting the risk of a single metabolic disorder or a single cardiovascular event, lacking systematic evaluations of these markers in the context of CMM, where multiple metabolic and cardiovascular conditions coexist. Moreover, the majority of these studies are based on specific populations or limited sample sizes, with insufficient evidence derived from large-scale, representative population-based databases. Therefore, the present study aims to investigate the accuracy, applicability, and comparative performance of various surrogate markers of IR under the condition of multimorbidity to provide more robust, evidence-based support for the early CMM identification. This study utilizes large-scale epidemiological data through a cross-sectional design to unveil the correlation of 5 IR surrogates (the TyG index, TyG-BMI index, METS-IR, TG/HDL-C ratio, and HOMA-IR) with CMM. This research aims to identify the optimal IR surrogate marker, provide scientific evidence for the early screening of CMM, and offer a basis for the formulation of public health intervention strategies and the early screening of CMM in the American population.

## 2. Methods

### 2.1. Study population and design

The National Health and Nutrition Examination Survey (NHANES) was utilized to gather baseline data. This database from the National Center for Health Statistics (NCHS) is an ongoing program and employs a complex, multi-stage sampling design to provide nationally representative, continuous cross-sectional surveys. The analysis results were adjusted using a weighting method to reflect the true distribution of the overall U.S. population (File S1, Supplemental Digital Content, https://links.lww.com/MD/R546). It primarily focuses on the health and nutrition of adults and children residing in America and enables a thorough assessment of the health of Americans. The NCHS gathers data every 2 years, including detailed information on medical history, laboratory results, physical examinations, nutritional intake, and demographics. For our study, data from 7 cycles of NHANES (2005–2018) were collected. This study focused on individuals who were older than 20 years, had CMM, and provided complete baseline data on TyG index, TyG-BMI index, METS-IR, TG/HDL-C ratio, and HOMA-IR. These data were used to examine the association between IR and the prevalence of CMM. The created and examined datasets can be accessed at http://www.cdc.gov/nchs/nhanes.htm. About 70,190 participants were encompassed for unraveling the link of 5 surrogate markers to IR, namely the TyG index, TyG-BMI index, METS-IR, TG/HDL-C ratio, and HOMA-IR, and CMM. The following were excluded from the study: those lacking relevant data for calculating IR surrogates, including fasting triglycerides, HDL, fasting plasma glucose, fasting serum insulin, and BMI; and those lacking data on CMM. Patients with no important covariates like gender, race, marital status, poverty-to-income ratio (PIR), education, and smoking. Patients with missing data on other laboratory covariates. Patients with fasting sub-sample weight (WTSAF2YR) ≤ 0. Ultimately, 15,537 participants were eligible (Figure S1, Supplemental Digital Content, https://links.lww.com/MD/R546). This study strictly adheres to the Strengthening the Reporting of Observational Studies in Epidemiology guidelines and has been approved by the NCHS Research Ethics Review Board, with all participants providing written informed consent.

### 2.2. IR surrogate indices

The IR surrogate indices were the TyG index, TyG-BMI index, METS-IR, TG/HDL-C ratio, and HOMA-IR. The TyG index is calculated through TyG index = ln**[**triglycerides (mg/dL) × glycemia (mg/dL)/2].^[10]^ The TyG-BMI index is derived as follows: TyG-BMI = TyG index × BMI (kg/m^2^).^[12]^ The METS-IR calculation is: ln[2 × glycemia (mg/dL) + triglycerides (mg/dL)] × BMI/ln[HDL-C (mg/dL)].^[13]^ Triglycerides (mg/dL) divided by HDL-C (mg/dL) yielded the TG/HDL-C ratio.^[14]^ The following is how HOMA-IR is calculated: fasting insulin (µU/mL) × fasting glucose (mmol/L)/22.5.^[9]^

### 2.3. Determination of CMM

CMM is the outcome measure of this study. The definition of CMM is the presence of 2 or more medical histories of hypertension, diabetes, coronary artery disease, or stroke.^[1]^ Specifically, stroke, CHD, hypertension, and diabetes were chosen because they represent the predominant contributors to the cardiometabolic disease burden in the US population. Furthermore, all 4 conditions are directly linked to IR through shared mechanisms, including endothelial dysfunction, chronic inflammation, and atherosclerosis. Data were obtained through participant interviews. Further details are available at http://www.cdc.gov/nchs/nhanes.htm. Any of the following criteria can be used to identify hypertension: self-reported hypertension, use of prescription antihypertensive drugs, or 3 consecutive resting readings with an average systolic blood pressure ≥ 140 mm Hg or an average diastolic blood pressure ≥ 90 mm Hg. With a threshold of 140/90 mm Hg for hypertension diagnosis, the standard is based on the International Society of Hypertension’s standards.^[17]^ Diabetes was confirmed based on the following criteria, with the presence of any one of them being sufficient for diagnosis: a positive response to the question “Are you taking medication to lower blood sugar?” is considered as anti-diabetic treatment; fasting plasma glucose ≥ 7 mmol/L or HbA1c ≥ 6.5% and/or self-reported anti-diabetic medication use defines diabetes.^[18]^ Information on CAD was obtained via inquiry: “Has a doctor or other health professional told you that you have CHD/angina/myocardial infarction?” Those answering “yes” to any of the above questions were diagnosed with CAD.^[19]^ Similarly, stroke information was obtained through self-reporting of a physician’s diagnosis of stroke.

### 2.4. Covariate assessment

The NHANES staff collected various relevant data through questionnaires administered during household interviews, including demographic information, medical history, and lab blood test results. Demographic variables encompassed age, sex, race, household income, marital status, education, and smoking. Race was divided into non-Hispanic Black, non-Hispanic White, Mexican American, other Hispanic, or other. According to PIR, household income was separated into low income (<1.0), moderate income (1.0–3.0), and high income (>3.0). There were 2 categories for marital status: with and without a partner. Education involved 2 categories: college and above, and high school or less. A person’s lifetime cigarette consumption was classified as either never smoking (<100 cigarettes), former smoking (over 100 cigarettes but more than 12 months of cessation), or current smoking (more than 100 cigarettes but either still smoking or had stopped smoking within 12 months).^[20]^ Laboratory blood test data encompassed levels of low-density lipoprotein cholesterol (LDL-C), high-density lipoprotein cholesterol (HDL-C), total cholesterol (TC), alanine aminotransferase (ALT), albumin, globulin, alkaline phosphatase (ALP), aspartate transaminase (AST), serum urea nitrogen, lactate dehydrogenase (LDH), serum creatinine (Scr), gamma-glutamyl transferase (GGT), total bilirubin (TBiL), uric acid, total protein, and hemoglobin.

### 2.5. Statistical analysis

Since our sample consists of fasting sub-samples, statistical weighting was applied to explain the NHANES survey design. The dependent variable was the participants’ existence of CMM, and laboratory markers and study population characteristics were stratified according to this. While categorical data were displayed as frequencies and percentages, continuous variables with normal and skewed distributions were reported as means ± standard deviations and medians (interquartile ranges). The *t* test and the Mann–Whitney *U* test were employed to identify group differences for continuous variables with normal and non-normal distributions. Baseline characteristics of categorical variables among cohorts were compared via the Pearson chi-square test.

Feature selection is a crucial stage in the model-building process. Boruta algorithm is a random forest-based supervised classification feature selection technique, which minimizes errors in random forest models and ultimately identifies the smallest optimal subset of features.^[21,22]^ Therefore, our study used the Boruta algorithm to determine all relevant features. In regression analysis, the TyG index and the 4 logarithmically transformed alternative IR indices (TyG-BMI index, METS-IR, TG/HDL-C ratio, and HOMA-IR) were continuous variables and classified into quartiles for analysis as categorical variables. The relation of surrogate IR indices to CMM was 1st assessed using multivariable logistic regression analysis, and the findings were displayed as odds ratios (OR) and 95% confidence intervals (CIs). Three regression models were built to control for confounding bias by adjusting for different covariates: Model 1 represents univariate logistic regression; Model 2 adjusts for age, gender, race, education, household income, as well as marital status; Model 3 adds laboratory continuous variables for a full adjustment based on Model 2. The nonlinear correlations of surrogate IR indicators with CMM were then determined through a restricted cubic spline (RCS) regression model. Additionally, receiver operating characteristic (ROC) curves and the area under the curve (AUC) were utilized for assessing the discriminative ability of various IR surrogates to predict CMM occurrence. AUC values were from 0 to 1, where higher values suggest stronger discrimination. The Brier score incorporates both model discrimination and calibration to evaluate overall model performance. A Brier score closer to 0 indicates that the predicted probabilities are more consistent with the actual outcomes.^[23]^ Lastly, subgroup analyses and interaction tests were undertaken based on PIR, smoking, age, gender, race, education, and marital status. For all statistical studies, R 4.4.1 (The R Foundation for Statistical Computing, Vienna, Austria) was used. *P* < .05 signified statistical significance.

## 3. Results

### 3.1. Baseline characteristics of study participants

The baseline characteristics are detailed in Table 1. About 15,537 participants were selected in this study. Regarding demographics, CMM patients exhibited a notably higher age (64 vs 44) and male percentage (52% vs 48%). Additionally, there were differences in racial distribution between the groups, with a higher proportion of non-Hispanic Black participants in the CMM group (14% vs 11%), while patients without CMM had more Mexican Americans (8.9% vs 6.4%). The proportion of non-Hispanic White participants was the same in both groups (67% vs 67%). Significant differences (*P* < .001) were also observed across baseline factors such as educational level, household income, and smoking history. The CMM group had a higher proportion of participants with lower educational levels, lower or middle household income, and a history of smoking. Regarding laboratory data, the CMM group showed higher levels in several blood biomarkers, including globulin, ALT, AST, ALP, serum urea nitrogen, Scr, GGT, LDH, and uric acid (all *P* < .001). In terms of predictor indicators, the CMM group also had markedly higher values for TyG index, METS-IR, TG/HDL-C ratio, HOMA-IR, and TyG-BMI index in contrast to the non-CMM cohort (*P* < .05).

**Table 1:** General characteristics of the participants.

Characteristic	N*	Overall	No CMM	CMM	*P*-value‡
		N = 215,754,164†	N = 185,475,046†	N = 30,279,117†	
Gender	15,537				**<.001**
Male		7487 (48%)	5971 (48%)	1516 (52%)	
Female		8050 (52%)	6685 (52%)	1365 (48%)	
Age	15,537	46 (33–60)	44 (31–56)	64 (55–73)	**<.001**
Age_group	15,537				**<.001**
<40		5156 (37%)	5046 (42%)	110 (4.9%)	
40–59		5194 (37%)	4426 (38%)	768 (32%)	
≥60		5187 (26%)	3184 (20%)	2003 (63%)	
Race	15,537				**<.001**
Mexican American		2499 (8.6%)	2120 (8.9%)	379 (6.4%)	
Other Hispanic		1579 (5.7%)	1295 (5.8%)	284 (4.9%)	
Non-Hispanic White		6593 (67%)	5354 (67%)	1239 (67%)	
Non-Hispanic Black		3081 (11%)	2354 (11%)	727 (14%)	
Other/multiracial		1785 (7.8%)	1533 (7.8%)	252 (7.4%)	
Education	15,537				**<.001**
High school and below		7394 (40%)	5721 (38%)	1673 (51%)	
Above high school		8143 (60%)	6935 (62%)	1208 (49%)	
Marital	15,537				.919
Having a partner		9514 (64%)	7784 (64%)	1730 (64%)	
Without partner		6023 (36%)	4872 (36%)	1151 (36%)	
PIR	15,537				**<.001**
<1.0		3231 (15%)	2574 (14%)	657 (17%)	
1.0–3.0		6559 (37%)	5188 (36%)	1371 (45%)	
≥3.0		5747 (49%)	4894 (50%)	853 (39%)	
Smoke	15,537				**<.001**
Never		8663 (55%)	7333 (57%)	1330 (45%)	
Former		3830 (25%)	2784 (23%)	1046 (38%)	
Current		3044 (20%)	2539 (20%)	505 (18%)	
Albumin (g/L)	15,537	42 (40–45)	43 (40–45)	41 (39–44)	**<.001**
ALT (IU/L)	15,537	21 (16–28)	21 (16–28)	22 (16–30)	**<.001**
AST (U/L)	15,537	22 (19–27)	22 (19–27)	23 (19–28)	**<.001**
ALP (IU/L)	15,537	65 (53–79)	64 (53–78)	70 (57–88)	**<.001**
Serum urea nitrogen (mmol/L)	15,537	4.64 (3.57–5.71)	4.28 (3.57–5.36)	5.36 (4.28–7.14)	**<.001**
Scr (µmol/L)	15,537	74 (64–88)	73 (63–86)	80 (66–97)	**<.001**
GGT (IU/L)	15,537	19 (14–29)	19 (13–28)	24 (17–37)	**<.001**
LDH (IU/L)	15,537	128 (113–146)	126 (112–144)	136 (118–156)	**<.001**
TBil (µmol/L)	15,537	12.0 (8.6–13.7)	12.0 (8.6–13.7)	10.3 (8.6–13.7)	**.018**
Total protein (g/L)	15,537	71.0 (68.0–74.0)	71.0 (68.0–74.0)	70.0 (68.0–74.0)	**.041**
Uric acid (µmol/L)	15,537	321 (268–375)	315 (262–375)	345 (286–410)	**<.001**
Globulin (g/L)	15,537	28 (26–31)	28 (26–31)	29 (26–32)	**<.001**
Hemoglobin (g/L)	15,537	14.40 (13.40–15.40)	14.40 (13.40–15.40)	14.10 (13.00–15.20)	**<.001**
TC (mmol/L)	15,537	190 (164–217)	192 (166–218)	175 (150–207)	**<.001**
LDL-C (mmol/L)	15,537	111 (89–135)	113 (92–137)	96 (75–123)	**<.001**
TyG index	15,537	8.55 (8.13–8.99)	8.49 (8.08–8.90)	8.99 (8.56–9.45)	**<.001**
METS-IR	15,537	41 (34–50)	40 (33–48)	49 (41–59)	**<.001**
TG/HDL-C ratio	15,537	1.92 (1.16–3.24)	1.81 (1.12–3.04)	2.67 (1.63–4.23)	**<.001**
HOMA-IR	15,537	2.33 (1.40–4.06)	2.14 (1.33–3.59)	4.36 (2.42–7.39)	**<.001**
TyG-BMI index	15,537	240 (201–288)	235 (197–279)	283 (235–337)	**<.001**

The data was shown as mean (SD) for continuous, n (%) for categorical.

ALP = alkaline phosphatase, ALT = alanine transaminase, AST = aspartate transaminase, CMM = cardiometabolic multimorbidity, GGT = gamma-glutamyltransferase, HOMA-IR = homeostasis model assessment of insulin resistance, LDH = lactate dehydrogenase, LDL-C = low-density lipoprotein cholesterol, METS-IR = metabolic score for insulin resistance, PIR = poverty-to-income ratio, Scr = serum creatinine, TBil = total bilirubin, TC = triglyceride, TG/HDL-C ratio = triglyceride/high-density lipoprotein cholesterol ratio, TyG index = Triglyceride-glucose index, TyG-BMI Index = triglyceride-glucose-body mass index.

* N not missing (unweighted).

† Median (Q1, Q3); n (unweighted) (%).

‡ Design-based Kruskal–Wallis test; Pearson χ^2^: Rao & Scott adjustment, *P* values in bold meant significantly different (*P* < .05).

### 3.2. Boruta feature selection

**Figure 1.**
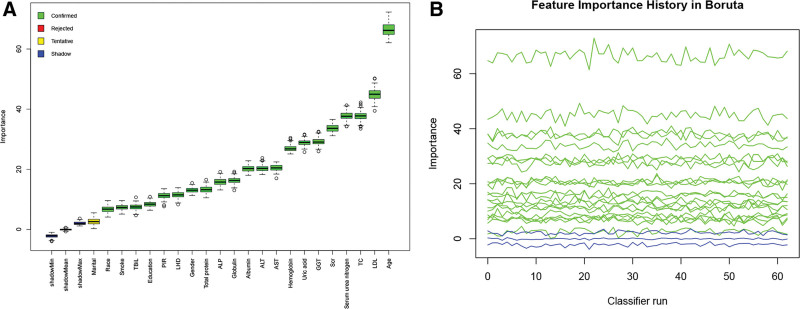
Feature selection process for CMM based on the Boruta algorithm (A) and the evolution of *Z*-score values during the selection process (B). In panel (A), the *x*-axis represents variable names, and the *y*-axis indicates the *Z*-scores of each variable. In panel (B), the *x*-axis denotes the number of iterations, while the *y*-axis illustrates the changes in *Z*-scores during the selection process. Blue boxes and lines correspond to the minimum, mean, and maximum *Z*-scores of shadow features. Green boxes and lines represent confirmed variables, while yellow boxes and lines indicate variables in an uncertain state. ALP = alkaline phosphatase; ALT = alanine transaminase; AST = aspartate transaminase; GGT = gamdma-glutamyl transferase; LDH = lactate dehydrogenase; LDL-C = low-density lipoprotein cholesterol; PIR = poverty-to-income ratio; TBil = total bilirubin; TC =triglycerides; Scr = serum creatinine.

Figure 1A, B presents the results of feature selection via the Boruta algorithm. After 500 iterations, the variables most strongly linked to CMM were identified, including age, LDL, TC, serum urea nitrogen, Scr, GGT, uric acid, hemoglobin, AST, ALT, albumin, globulin, ALP, total protein, gender, LDL, PIR, education, TBiL, smoking history, and race. Furthermore, marital status was not found to have a significantly higher importance score compared to the strongest associated features or shadow features and was thus considered uncertain. However, based on prior research and clinical experience, the following studies continued to take marital status into account.

### 3.3. The relationship between IR surrogates and CMM

Three weighted logistic regression models unraveled the link of TyG index, TyG-BMI index, METS-IR, TG/HDL-C ratio, HOMA-IR, to CMM, as presented in Table 2. Variables adjusted in Model 3 were selected according to the results of the Boruta algorithm for feature selection and included age, sex, household income, education, marital status, smoking, LDL cholesterol, TC, hemoglobin, globulin, uric acid, total protein, TBiL, LDH, GGT, Scr, serum urea nitrogen, ALP, AST, ALT, and albumin. The analysis demonstrated that when treated as continuous variables, all 5 IR surrogates were positively associated with CMM in both Model 1 (unadjusted) and Model 2 (adjusted for age, sex, race, education, marital status, and household income), with all associations achieving statistical significance (*P* < .001). Similarly, in the fully adjusted Model 3, the following positive associations with CMM remained statistically significant: TyG index (OR = 4.857, 95% CI [4.159–5.67], *P* < .001); ln[TyG-BMI] (OR = 26.34, 95% CI [19.08–36.34], *P* < .001); ln[METS-IR] (OR = 21.31, 95% CI [15.56–29.19], *P* < .001); ln[TG/HDL-C] (OR = 2.05, 95% CI [1.853–2.267], *P* < .001), and ln[HOMA-IR] (OR = 2.92, 95% CI [2.622–3.251], *P* < .001). When analyzed as categorical variables, these 5 surrogates also exhibited significant positive correlations with the risk of CMM across all 3 weighted multivariable logistic regression models (*P* < .001). Furthermore, trend tests for the quartiles of these surrogates revealed significant trend effects (*P* for trend < .05) (Table 2). In comparison to individuals in the lowest quartile (Q1), those in higher quartiles (Q2/Q3/Q4) showed a progressively increased risk of CMM.

**Table 2:** Associations between different IR surrogates and CMM.

	Model 1*			Model 2†			Model 3‡		
	OR	95% CI	*P*	OR	95% CI	*P*	OR	95% CI	*P*
TyG index	3.238	2.932–3.576	**<.001**	3.283	2.930–3.679	**<.001**	4.857	4.159–5.673	**<.001**
Categories									
<8.13	–	–		–	–		–	–	
8.13–8.55	2.025	1.651–2.483	**<.001**	1.558	1.242–1.953	**<.001**	1.859	1.478–2.339	**<.001**
8.55–8.19	3.38	2.810–4.066	**<.001**	2.559	2.085–3.140	**<.001**	3.419	2.764–4.228	**<.001**
≥8.19	7.911	6.507–9.616	**<.001**	6.204	5.022–7.663	**<.001**	9.238	7.260–11.76	**<.001**
*P* for trend	1.993	1.875–2.118	**<.001**	1.904	1.782–2.034	**<.001**	2.141	1.986–2.308	**<.001**
TyG-BMI index	15.65	12.17–20.13	**<.001**	24.15	17.87–32.62	**<.001**	26.34	19.08–36.34	**<.001**
Categories									
<201.03	–	–		–	–		–	–	
201.03–240.10	2.013	1.592–2.545	**<.001**	1.423	1.142–1.774	**.002**	1.677	1.344–2.093	**<.001**
240.10–287.63	2.892	2.353–3.555	**<.001**	2.14	1.761–2.600	**<.001**	2.531	2.066–3.100	**<.001**
≥287.63	6.74	5.509–8.246	**<.001**	6.293	5.182–7.642	**<.001**	6.796	5.493–8.407	**<.001**
*P* for trend	1.869	1.761–1.984	**<.001**	1.933	1.812–2.062	**<.001**	1.929	1.799–2.070	**<.001**
METS-IR	12.31	9.680–15.66	**<.001**	21.2	15.92–28.23	**<.001**	21.31	15.56–29.19	**<.001**
Categories									
<33.74	–	–		–	–		–	–	
33.74–41.03	1.864	1.485–2.341	**<.001**	1.439	1.151–1.797	**.002**	1.617	1.283–2.038	**<.001**
41.03–49.9	3.071	2.509–3.760	**<.001**	2.568	2.114–3.118	**<.001**	2.842	2.299–3.513	**<.001**
≥49.9	6.414	5.173–7.954	**<.001**	6.822	5.465–8.516	**<.001**	6.778	5.312–8.649	**<.001**
*P* for trend	1.858	1.750–1.972	**<.001**	1.99	1.860–2.129	**<.001**	1.942	1.801–2.094	**<.001**
TG/HDL-C ratio	1.818	1.687–1.959	**<.001**	1.986	1.807–2.181	**<.001**	2.05	1.853–2.267	**<.001**
Categories									
<1.16	–	–		–	–		–	–	
1.16–1.92	1.566	1.332–1.840	**<.001**	1.522	1.294–1.789	**<.001**	1.632	1.372–1.942	**<.001**
1.92–3.64	2.397	2.015–2.851	**<.001**	2.26	1.870–2.731	**<.001**	2.355	1.921–2.887	**<.001**
≥3.64	3.581	3.029–4.234	**<.001**	3.867	3.212–4.656	**<.001**	4.147	3.400–5.058	**<.001**
*P* for trend	1.526	1.449–1.606	**<.001**	1.571	1.479–1.669	**<.001**	1.594	1.498–1.697	**<.001**
HOMA-IR	2.813	2.557–3.094	**<.001**	2.923	2.630–3.248	**<.001**	2.92	2.622–3.251	**<.001**
Categories									
<1.34	–	–		–	–		–	–	
1.34–2.33	1.31	1.066–1.610	**.011**	1.129	0.907–1.406	.273	1.186	0.948–1.484	.133
2.33–4.06	2.522	2.043–3.113	**<.001**	2.078	1.685–2.564	**<.001**	2.215	1.780–2.755	**<.001**
≥4.06	7.031	5.685–8.697	**<.001**	6.525	5.301–8.032	**<.001**	6.48	5.168–8.125	**<.001**
*P* for trend	2.062	1.925–2.209	**<.001**	2.038	1.901–2.185	**<.001**	2.006	1.865–2.156	**<.001**

*P* values in bold meant significantly different (*P* < .05).

ALP = alkaline phosphatase, ALT = alanine transaminase, AST = aspartate transaminase, BMI = body mass index, CIs = confidence intervals, CMM = cardiometabolic multimorbidity, GGT = gamma-glutamyltransferase, HOMA-IR = homeostasis model assessment of insulin resistance, LDH = lactate dehydrogenase, LDL-C = low-density lipoprotein cholesterol, METS-IR = metabolic score for insulin resistance, OR = odds ratio, PIR = poverty-to-income ratio, Scr = serum creatinine, TBil = total bilirubin, TC = triglyceride, TG/HDL-C ratio = triglyceride/high-density lipoprotein cholesterol ratio, TyG index = triglyceride-glucose index, TyG-BMI index = triglyceride-glucose-body mass index.

* Model 1 was crude model.

† Model 2 was adjusted for age, race, gender, educational level, marital status PIR.

‡ Model 3 was adjusted for age, race, gender, educational level, marital status, PIR, smoking, albumin, globulin, ALT, AST, ALP, serum urea nitrogen, Scr, GGT, LDH, uric acid, hemoglobin, TC, LDL-C, TBil, total protein.

### 3.4. Detection of nonlinear relationships

**Figure 2.**
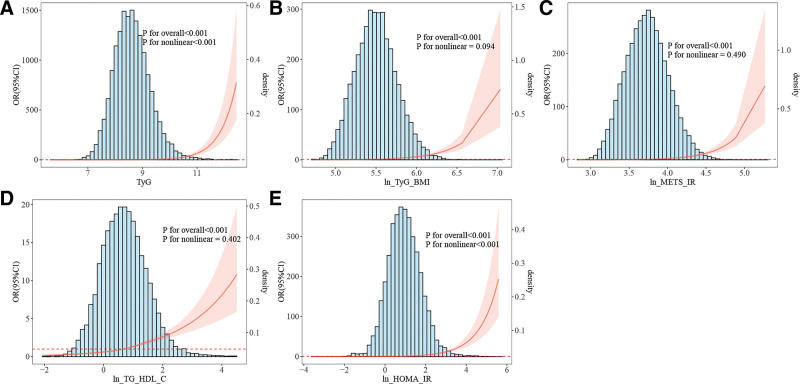
RCS analysis of the association between different insulin resistance (IR) surrogates and CMM. (A) Association between TyG index and CMM; (B) association between ln[TyG_BMI] and CMM; (C) association between ln[METS_IR] and CMM; (D) association between ln[TG-HDL-C] and CMM; (E) association between ln[HOMA_IR] and CMM. CMM = cardiometabolic multimorbidity; HOMA-IR = homeostasis model assessment of insulin resistance; METS-IR = metabolic score for insulin resistance; RCS = restricted cubic spline; TG/HDL-C ratio = triglyceride/high-density lipoprotein cholesterol ratio; TyG-BMI index = triglyceride glucose-body mass index; TyG index = triglyceride-glucose index.

Given that weighted multivariable logistic regression analysis indicated potential nonlinear associations between the 5 surrogate markers and CMM, to unveil these correlations further, a weighted RCS analysis was completed. As shown in Figure 2A and E, the relations of the TyG index and ln[HOMA-IR] to CMM demonstrated a nonlinear “J-shaped” pattern (*P* for overall < .001, *P* for nonlinear < .05). Conversely, ln[TyG-BMI], ln[METS-IR], and ln[TG/HDL-C] exhibited significant linear relationships with CMM risk (*P* for overall < .001, *P* for nonlinear > .05) (Fig. 2B–D).

### 3.5. Subgroup analyses

**Figure 3.**
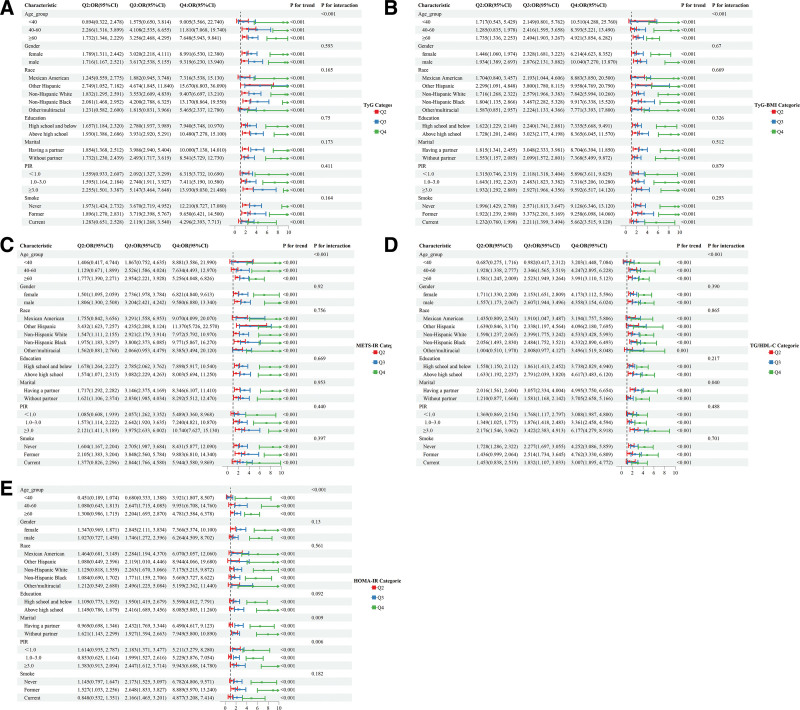
Subgroup analysis of the association between different IR surrogates and CMM. (A) TyG index; (B) TyG-BMI index; (C) METS-IR; (D) TG/HDL-C ratio; (E) HOMA-IR. CI = confidence interval; CMM = cardiometabolic multimorbidity; HOMA-IR = homeostasis model assessment of insulin resistance; METS-IR = metabolic score for insulin resistance; OR = odds ratio; PIR = poverty-to-income ratio; TG/HDL-C ratio = triglyceride/high-density lipoprotein cholesterol ratio; TyG-BMI index = triglyceride-glucose-body mass index; TyG index = triglyceride-glucose index.

Subgroup analyses investigated the effects of various factors like age, sex, race, education, marital status, household income, and smoking on the link of TyG index, TyG-BMI index, METS-IR, TG/HDL-C ratio, HOMA-IR, to CMM risk. As illustrated in Figure 3A–E and detailed in Tables S1–S5, Supplemental Digital Content, https://links.lww.com/MD/R546, the relationships of all 5 IR surrogates with CMM were influenced by age (*P* for interaction < .05). Additionally, the relationships of TG/HDL-C ratio and HOMA-IR with CMM were significantly affected by marital status and PIR (*P* for interaction < .05), being more pronounced in the elderly, those without partners, and those with higher household incomes. By contrast, the associations of other IR surrogates with CMM remained robust across subgroups, except for age (*P* for interaction > .05).

### 3.6. Model evaluation and calibration analysis

**Figure 4.**
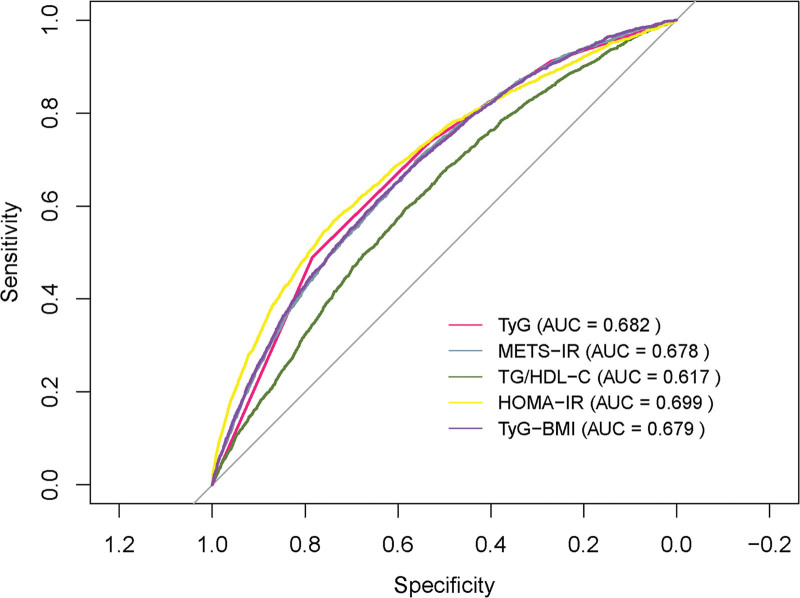
ROC curves for predicting CMM risk using different IR surrogates. AUC = area under the curve; CMM = cardiometabolic multimorbidity; HOMA-IR = homeostasis model assessment of insulin resistance; METS-IR = metabolic score for insulin resistance; ROC = receiver operating characteristic; TG/HDL-C ratio = triglyceride/high-density lipoprotein cholesterol ratio; TyG-BMI index = triglyceride glucose-body mass index; TyG index = triglyceride-glucose index.

**Figure 5.**
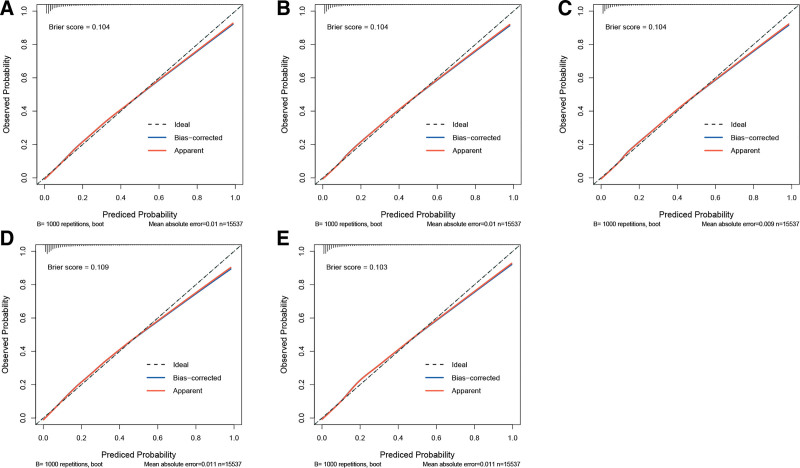
Calibration curve of the model. (A) TyG index; (B) TyG-BMI index; (C) METS-IR; (D) TG/HDL-C ratio; (E) HOMA-IR. HOMA-IR = homeostasis model assessment of insulin resistance; METS-IR = metabolic score for insulin resistance; TG/HDL-C ratio = triglyceride/high-density lipoprotein cholesterol ratio; TyG-BMI index = triglyceride-glucose-body mass index; TyG index = triglyceride-glucose index.

The discriminatory power of different IR surrogates was assessed for predicting CMM risk through ROC curve analysis. As shown in Figure 4, HOMA-IR demonstrated the highest AUC at 0.699, significantly outperforming the other 4 surrogates and indicating superior accuracy. The TyG index followed (AUC = 0.682), along with the TyG-BMI index (AUC = 0.679), METS-IR (AUC = 0.678), and TG/HDL-C ratio, which had the lowest AUC (AUC = 0.617). To comprehensively assess the predictive performance of the models, 5 surrogate indices of IR were further calculated. As shown in Figure 5A–E, the Brier scores for the TyG index, TyG-BMI index, METS-IR, TG/HDL-C ratio, and HOMA-IR were 0.104, 0.104, 0.104, 0.109, and 0.103, respectively.

## 4. Discussion

CMM, as a complex state of coexisting metabolic and cardiovascular diseases, is closely associated with IR.^[4]^ Our study elucidated the correlations between 5 IR surrogates (TyG index, TyG-BMI index, METS-IR, TG/HDL-C ratio, and HOMA-IR) and CMM in the American community population. Unlike previous studies that relied solely on clinical experience to select variables, results from the Boruta algorithm were integrated with traditional risk factors. Weighted multivariable logistic regression analysis revealed that higher levels of the foregoing 5 surrogates were notably related to a rising risk of CMM. RCS analysis demonstrated a nonlinear “J-shaped” relationship between the TyG index and HOMA-IR and CMM, whereas TyG-BMI index, METS-IR, and TG/HDL-C ratio exhibited linear relationships with CMM risk. Moreover, the discriminating power of unadjusted IR surrogates was assessed using ROC curves. The results showed that HOMA-IR performed best in predicting CMM risk, yielding an AUC of 0.699. This study provides valuable evidence through a systematic comparison of 5 IR surrogates, contributing to early screening and intervention strategies.

In our analysis, HOMA-IR outperformed the TyG index, TyG-BMI index, TG/HDL-C ratio, and METS-IR in predicting CMM. TyG index, obtained from triglyceride and fasting glucose levels, is a simple and practical surrogate that has demonstrated strong predictive ability in metabolic syndrome, nonalcoholic steatohepatitis, and cardiovascular diseases.^[24–26]^ While the TyG index showed clinical utility in predicting CMM (AUC = 0.682), its ability to capture complex metabolic disturbances is limited, as it primarily reflects disruptions in lipid and glucose metabolism without directly assessing insulin sensitivity. The TyG-BMI index, which combines BMI with the TyG index, enhances its capacity to reflect obesity-related metabolic abnormalities. Previous research highlighted the prognostic value of TyG-BMI in acute myocardial infarction patients.^[27]^ In this study, the TyG-BMI index achieved an AUC of 0.679, but it did not significantly outperform the TyG index or other surrogates. This may be attributed to the lack of specificity of BMI in some non-obese individuals, as the occurrence of CMM often involves multi-dimensional metabolic abnormalities.^[28]^ Additionally, while obesity is closely linked to IR, their relationship is not entirely linear,^[29,30]^ potentially limiting the applicability of the TyG-BMI index in certain contexts.

TG/HDL-C ratio, a traditional lipid metabolism indicator, has been broadly utilized to forecast the likelihood of cardiovascular events.^[31]^ However, the TG/HDL-C ratio achieved an AUC of only 0.617 for predicting CMM, which deviates from findings reported in other studies. Similarly, in studies involving patients with chronic heart failure,^[32]^ the TyG index exhibited superior predictive performance in contrast to TyG-BMI and TG/HDL-C ratio. This discrepancy may stem from TG/HDL-C ratio’s primary focus on reflecting lipid metabolism dysfunction, without capturing the dynamic changes in glucose metabolism and insulin function. Nevertheless, the TG/HDL-C ratio remains valuable in primary healthcare settings, especially in resource-limited environments.

METS-IR, as a composite score incorporating parameters such as BMI, triglycerides, and HDL-C, has been recognized for its suitability in forecasting the likelihood of diabetes^[33]^ and cardiovascular diseases.^[34]^ Research has demonstrated that METS-IR performed well in predicting adverse cardiovascular events in patients with metabolic syndrome and heart failure, with an AUC of 0.691.^[35]^ In this study, the predictive performance of METS-IR (AUC = 0.678) was comparable to previous findings but remained inferior to that of HOMA-IR (AUC = 0.699). This limitation may be attributed to METS-IR’s inability to adequately reflect changes in pancreatic β-cell function and insulin sensitivity, which greatly bear on the development of CMM.^[5]^

Unlike the aforementioned 4 indicators, HOMA-IR has the advantage of directly integrating fasting glucose and insulin, comprehensively reflecting both insulin sensitivity and pancreatic β-cell function.^[36,37]^ In our study, HOMA-IR demonstrated the highest predictive performance for CMM (AUC = 0.699), consistent with previous findings.^[38]^ Similarly, a study on metabolic dysfunction-associated steatotic liver disease^[39]^ reported that HOMA-IR significantly outperformed the TyG index (AUC = 0.630) and TG/HDL-C (AUC = 0.614). Furthermore, HOMA-IR has been strongly connected with the likelihood of developing atherosclerosis,^[40]^ and nonalcoholic fatty liver disease.^[41]^ Because of its high sensitivity in assessing dynamic changes in IR, HOMA-IR has also demonstrated significant value in long-term follow-up and evaluating the effectiveness of interventions.^[42]^ HOMA-IR, a model reflecting the relationship between fasting glucose and insulin dynamics, is commonly employed to estimate IR.^[43]^ In this study, HOMA-IR demonstrated the highest diagnostic accuracy. Nonetheless, its calculation relies on fasting serum insulin levels, which limits its utility in individuals with β-cell dysfunction or those receiving exogenous insulin therapy.^[44]^ Moreover, the requirement for fasting insulin measurement poses a challenge in resource-limited settings, where such assays are often unavailable or prohibitively expensive in primary care contexts. Under such circumstances, surrogate indices based solely on routine lipid and glucose measurements, such as the TyG index or METS-IR, possibly offer a pragmatic compromise between feasibility and diagnostic precision. However, in well-equipped medical centers or research settings, HOMA-IR remains a valuable and accurate tool for assessing IR. Future research should explore the development of point-of-care insulin assays or validate simplified formulas tailored for underserved populations.

The potential association between HOMA-IR and CMM can be explained through multiple mechanisms supported by international studies. IR, the core feature of HOMA-IR, drives the progression of CMM through metabolic and inflammatory pathways.^[45]^ IR reduces tissue sensitivity to insulin, resulting in hyperglycemia and further exacerbating lipid metabolism disturbances and energy imbalance.^[46,47]^ Additionally, IR is closely linked to chronic low-grade inflammation, which accelerates the pathological progression of CMM by inducing endothelial dysfunction and atherosclerosis.^[48,49]^ Research indicates that IR activates the nuclear factor κB signaling pathway, triggering the release of pro-inflammatory factors like interleukin-6 and tumor necrosis factor-α, thereby inducing systemic inflammatory responses. Moreover, immune dysregulation significantly impacts the relationship between IR and CMM. Disruption of insulin signaling may lead to excessive immune activation, enhancing the inflammatory phenotypes of monocytes and T cells.^[50]^ These immune and inflammatory factors collectively form the complex pathological basis of CMM. In summary, HOMA-IR reflects the interplay of multiple factors, including immunity,^[51]^ inflammation,^[52]^ and IR.^[29]^ It is proposed that HOMA-IR has a unique advantage in capturing these mechanisms, thereby providing a robust scientific basis for predicting CMM risk. Thus, HOMA-IR is not only an effective diagnostic tool but also an important resource for elucidating the pathological mechanisms underlying CMM.

Notably, the AUC values for IR-related surrogate indices in this study did not reach high levels, a finding consistent with prior high-quality studies in related fields. For instance, in a longitudinal analysis from the CARDIA cohort, the AUCs of the TyG index and HOMA-IR for predicting the risk of congestive heart failure in young adults were 0.67 and 0.675, respectively, with no statistically significant difference between the 2.^[53]^ Similarly, in a healthy Chinese population, 7 lipid- and obesity-related indicators for IR yielded AUCs mostly ranging between 0.6 and 0.7, with the best-performing index (TyG-BMI) achieving an AUC of only 0.729.^[54]^ In predicting the risk of metabolic syndrome among middle-aged and elderly individuals, most indices likewise yielded AUCs within the 0.6 to 0.75 range, with relatively better performance seen in TyG-BMI and CVAI, yet still falling short of 0.76.^[55]^ Among patients with type 2 diabetes mellitus, commonly used IR-related indicators such as TyG and TG/HDL-C also demonstrated only modest predictive accuracy for diabetic kidney disease, with AUCs mostly between 0.6 and 0.7.^[56]^ This moderate discriminative performance is primarily attributable to the highly multifactorial and heterogeneous etiology of complex metabolic disorders. Although IR-related indicators can reflect certain aspects of metabolic and cardiovascular risk, the development of conditions such as CMM, diabetic kidney disease, and metabolic syndrome is also influenced by genetic predisposition, inflammatory status, and lifestyle factors. Therefore, the discriminative capacity of any single biomarker is inherently limited. Additionally, IR surrogate indices themselves are susceptible to variability related to individual physiological states, timing of measurement, and methodological inconsistencies, further constraining the potential for improved AUC performance. Therefore, even in large-sample studies across diverse disease populations, these biomarkers consistently demonstrate only moderate discriminative ability, an observation that reflects both the multifactorial nature of metabolic disorders and the inherent limitations of single-indicator screening strategies. In the future, integrated predictive models incorporating multi-omics variables, such as inflammation, genetics, and behavioral factors, possibly enhance the risk assessment capabilities for complex conditions like CMM.

This study offers a comprehensive evaluation, using a large, nationally representative U.S. adult sample, of the associations between 5 IR surrogate indices and CMM risk in the general community population. To strengthen the validity of our findings, a variety of potential confounders were adjusted for in our models, including age, sex, race/ethnicity, marital status, household income, and numerous laboratory parameters, to minimize bias and improve reliability. Logistic regression analysis, combined with the Boruta algorithm, was employed to enhance the robustness and accuracy of the findings. Among the indices evaluated, HOMA-IR demonstrated the highest predictive performance for CMM events. These findings not only fill a critical gap in the literature but also provide novel insights for future research into IR-related surrogate markers.

### 4.1. Limitations

The limitations of our study are as follows. First, due to the inherent constraints of a cross-sectional study design, causal inferences between HOMA-IR and CMM remain challenging and require validation through large-scale, prospective cohort studies. Second, the findings are based on the American population, which may limit their generalizability to other ethnicities and populations. Furthermore, despite a systematic evaluation of various IR-related surrogate indices, none of the AUCs exceeded 0.7, consistent with findings from leading international studies, highlighting the limited discriminative power of single metabolic indicators in predicting the risk of complex chronic diseases. This phenomenon may be explained by the multifactorial pathophysiology of conditions such as CMM, inter-individual variability in IR-related markers, and technical limitations of biomarker measurement. Lastly, self-reporting is one of the most effective means to conveniently and quickly obtain information related to IR indicators and the incidence of CMM in NHANES participants. However, due to the limitations of the self-reporting method, this approach may inevitably lead to recall bias. Therefore, caution should be exercised when analyzing and interpreting data. Future research can further improve the external applicability and discriminative efficacy of risk models by designing prospective cohort studies, incorporating multi-dimensional indicators such as inflammation and genetics, and expanding multi-center populations.

## 5. Conclusion

This study systematically evaluates the relations of 5 IR surrogate indices to CMM. Among them, HOMA-IR demonstrated the highest predictive performance (AUC = 0.699) and exhibited a J-shaped association, while the other indices showed linear positive correlations with CMM risk. Despite the limitations of a cross-sectional design, our findings provide new evidence to support early CMM screening. Further longitudinal investigations are warranted to verify the causality of these associations and to explore the development of clinically applicable and simplified screening tools.

## Acknowledgements

We acknowledged the contributions of NHANES staff for creating and updating the NHANES database.

## Author contributions

**Conceptualization:** Youfu He, Lei Peng.

**Data curation:** Youfu He, Lei Peng.

**Funding acquisition:** Lei Peng.

**Investigation:** Youfu He, Lei Peng.

**Methodology:** Youfu He, Lei Peng.

**Writing – original draft:** Xiuxia Song, Youfu He, Zhonggui Cai, Lei Peng.

**Writing – review & editing:** Xiuxia Song, Youfu He, Zhonggui Cai, Lei Peng.

## Supplementary Material
