# Supplementary material for: Exploring the association between 5 different alternative indicators of insulin resistance and the risk of multiple cardiovascular and metabolic diseases: A cross-sectional NHANES study from 2005 to 2018
Source: Medicine (Baltimore). 2026 Mar 13;105(11):e48080. doi: 10.1097/MD.0000000000048080 (PMC12991457; doi:10.1097/MD.0000000000048080)
Supplement: Supplementary file 1 [file medi-105-e48080-s001.docx]

**Supplementary File 1**

To: **Exploring the association between five different alternative indicators of insulin resistance and the risk of multiple cardiovascular and metabolic diseases: A cross-sectional NHANES study from 2005 to 2018**

by Xiuxia Song, Youfu He, Zhonggui Cai, Lei Peng

**Supplementary Method**

***Weighted analysis***

Weighted analysis is a statistical method that assigns different weights to observations in a sample to reflect their importance and representativeness within the overall dataset. This technique enhances the accuracy and efficiency of estimates and allows for a deeper understanding of population characteristics and relationships between variables. By adjusting sample weights, weighted analysis corrects estimation biases, leading to more accurate results. Additionally, it optimizes estimation efficiency by assigning different weights to observations based on their relative importance, ensuring that the sample is representative and leading to more accurate population estimates[1]. Detailed information and methodology regarding the National Health and Nutrition Examination Survey (NHANES) weighting can be found in the NHANES guidelines on their website [1]. Following these guidelines, we incorporated the complex sampling design and mobile examination center sample weights into our study, ensuring that our data sample represents a large and diverse U.S. adult population.

**References:**

1. NHANES Survey Methods and Analytic Guidelines. [https://wwwn.cdc.gov/nchs/nhanes/analyticguidelines.aspx. Accessed 12 Aug 2024](https://wwwn.cdc.gov/nchs/nhanes/analyticguidelines.aspx.%20Accessed%2012%20Aug%202024)

Supplement Figure


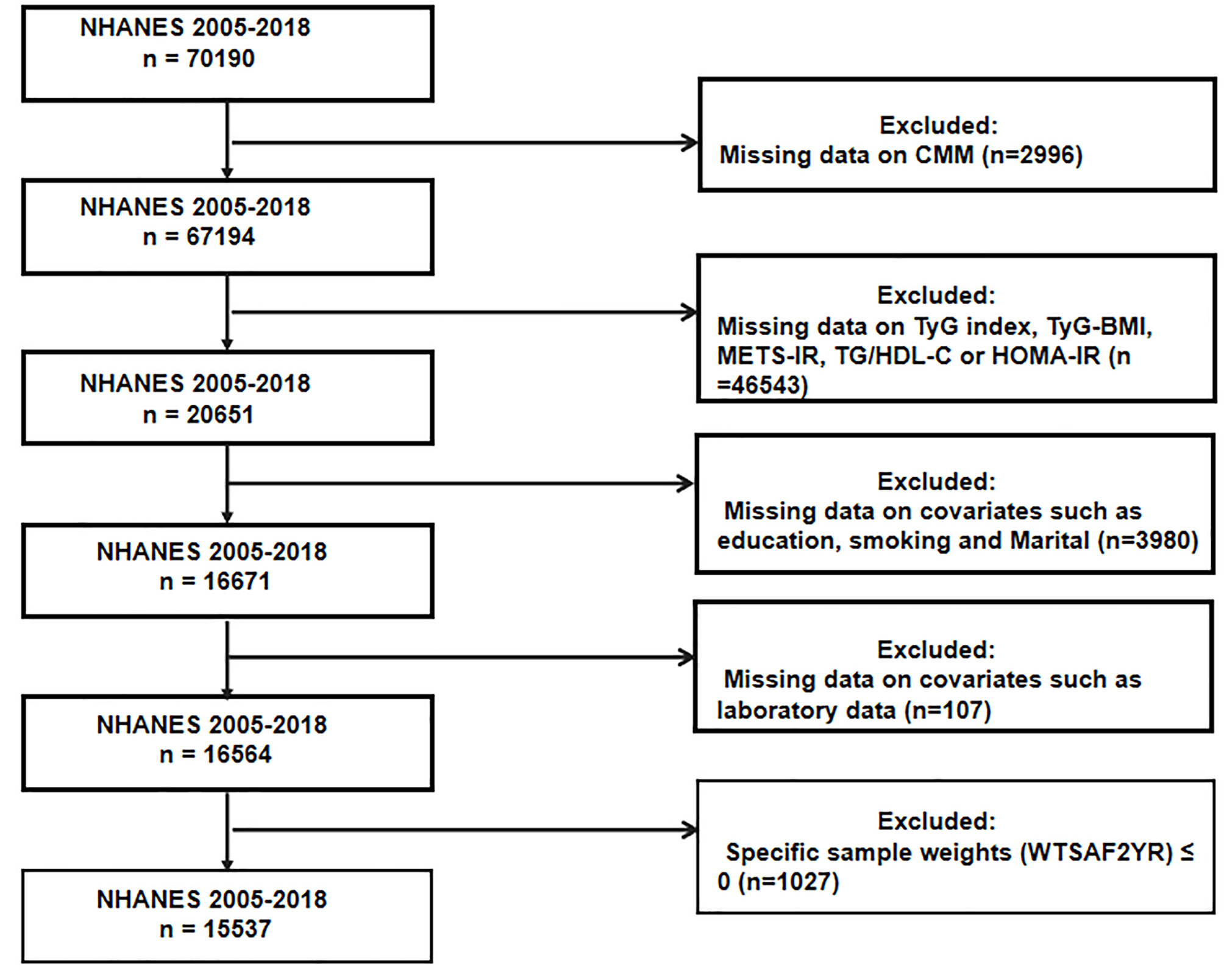


**Supplementary Figure 1**. Flowchart of Patient Selection.

**Abbreviations:** NHANES, the National Health and Nutrition Examination Survey; TyG Index, triglyceride-glucose index; METS-IR, metabolic score for insulin resistance; TyG-BMI Index, triglyceride-glucose body mass index; TG/HDL-C Ratio, triglyceride/high-density lipoprotein cholesterol ratio; HOMA-IR, homeostasis model assessment of insulin resistance

**Supplementary Table 1** Subgroup Analysis of the Association Between the TyG Index and CMM.

| **Characteristic** | **8.13-8.55** | | | **8.55-8.19** | | | **≥8.19** | | | **P for trend** | **P for interaction** |
| --- | --- | --- | --- | --- | --- | --- | --- | --- | --- | --- | --- |
|  | **OR** | **lower** | **upper** | **OR** | **lower** | **upper** | **OR** | **lower** | **upper** |  |  |
| Age_group |  |  |  |  |  |  |  |  |  |  | <0.001 |
| <40 | 0.894 | 0.322 | 2.478 | 1.575 | 0.65 | 3.814 | 9.005 | 3.566 | 22.74 | <0.001 |  |
| 40-60 | 2.266 | 1.316 | 3.899 | 4.108 | 2.535 | 6.655 | 11.81 | 7.068 | 19.74 | <0.001 |  |
| ≥60 | 1.732 | 1.346 | 2.229 | 3.256 | 2.468 | 4.295 | 7.648 | 5.943 | 9.841 | <0.001 |  |
| Gender |  |  |  |  |  |  |  |  |  |  | 0.593 |
| female | 1.789 | 1.311 | 2.442 | 3.02 | 2.218 | 4.111 | 8.991 | 6.53 | 12.38 | <0.001 |  |
| male | 1.716 | 1.167 | 2.521 | 3.617 | 2.538 | 5.155 | 9.319 | 6.23 | 13.94 | <0.001 |  |
| Race |  |  |  |  |  |  |  |  |  |  | 0.165 |
| Mexican American | 1.245 | 0.559 | 2.775 | 1.882 | 0.945 | 3.748 | 7.316 | 3.538 | 15.13 | <0.001 |  |
| Other Hispanic | 2.749 | 1.052 | 7.182 | 4.674 | 1.845 | 11.84 | 15.67 | 6.803 | 36.09 | <0.001 |  |
| Non-Hispanic White | 1.832 | 1.295 | 2.591 | 3.553 | 2.609 | 4.839 | 9.407 | 6.697 | 13.21 | <0.001 |  |
| Non-Hispanic Black | 2.081 | 1.468 | 2.952 | 4.2 | 2.788 | 6.325 | 13.17 | 8.864 | 19.55 | <0.001 |  |
| Other/multiracial | 1.231 | 0.582 | 2.6 | 1.815 | 0.831 | 3.966 | 5.465 | 2.337 | 12.78 | <0.001 |  |
| Education |  |  |  |  |  |  |  |  |  |  | 0.75 |
| High school and below | 1.657 | 1.184 | 2.32 | 2.78 | 1.937 | 3.989 | 7.94 | 5.748 | 10.97 | <0.001 |  |
| Above high school | 1.93 | 1.386 | 2.686 | 3.931 | 2.92 | 5.291 | 10.48 | 7.278 | 15.1 | <0.001 |  |
| Marital |  |  |  |  |  |  |  |  |  |  | 0.173 |
| Having a partner | 1.854 | 1.368 | 2.512 | 3.986 | 2.94 | 5.404 | 10 | 7.138 | 14.01 | <0.001 |  |
| Without partner | 1.732 | 1.23 | 2.439 | 2.493 | 1.717 | 3.619 | 8.541 | 5.729 | 12.73 | <0.001 |  |
| PIR |  |  |  |  |  |  |  |  |  |  | 0.411 |
| <1.0 | 1.559 | 0.933 | 2.607 | 2.092 | 1.327 | 3.299 | 6.315 | 3.732 | 10.69 | <0.001 |  |
| 1.0–3.0 | 1.595 | 1.164 | 2.184 | 2.74 | 1.911 | 3.927 | 7.411 | 5.19 | 10.58 | <0.001 |  |
| ≥3.0 | 2.255 | 1.501 | 3.387 | 5.147 | 3.464 | 7.648 | 13.93 | 9.03 | 21.48 | <0.001 |  |
| Smoke |  |  |  |  |  |  |  |  |  |  | 0.164 |
| Never | 1.973 | 1.424 | 2.732 | 3.67 | 2.719 | 4.952 | 12.21 | 8.727 | 17.08 | <0.001 |  |
| Former | 1.896 | 1.27 | 2.831 | 3.719 | 2.398 | 5.767 | 9.65 | 6.421 | 14.5 | <0.001 |  |
| Current | 1.283 | 0.651 | 2.528 | 2.119 | 1.268 | 3.54 | 4.296 | 2.393 | 7.713 | <0.001 |  |

**Abbreviations:** OR, Odds Ratio; PIR, Income -poverty ratio; TyG index, triglyceride glucose index; CMM, cardiometabolic multimorbidity

**Supplementary Table 2** Subgroup Analysis of the Association Between the TyG-BMI Index and CMM.

| **Characteristic** | **201.03-240.10** | | | **240.10-287.63** | | | **≥287.63** | | | **P for trend** | **P for interaction** |
| --- | --- | --- | --- | --- | --- | --- | --- | --- | --- | --- | --- |
|  | **OR** | **lower** | **upper** | **OR** | **lower** | **upper** | **OR** | **lower** | **upper** |  |  |
| Age_group |  |  |  |  |  |  |  |  |  |  | <0.001 |
| <40 | 1.717 | 0.543 | 5.429 | 2.149 | 0.801 | 5.762 | 10.51 | 4.288 | 25.76 | <0.001 |  |
| 40-60 | 1.285 | 0.835 | 1.978 | 2.416 | 1.595 | 3.658 | 8.393 | 5.221 | 13.49 | <0.001 |  |
| ≥60 | 1.735 | 1.336 | 2.253 | 2.494 | 1.903 | 3.267 | 4.921 | 3.854 | 6.282 | <0.001 |  |
| Gender |  |  |  |  |  |  |  |  |  |  | 0.67 |
| female | 1.446 | 1.06 | 1.974 | 2.328 | 1.681 | 3.223 | 6.214 | 4.623 | 8.352 | <0.001 |  |
| male | 1.934 | 1.389 | 2.693 | 2.876 | 2.131 | 3.882 | 10.04 | 7.27 | 13.87 | <0.001 |  |
| Race |  |  |  |  |  |  |  |  |  |  | 0.669 |
| Mexican American | 1.704 | 0.84 | 3.457 | 2.193 | 1.044 | 4.606 | 8.883 | 3.85 | 20.5 | <0.001 |  |
| Other Hispanic | 2.299 | 1.091 | 4.848 | 3.8 | 1.78 | 8.115 | 9.958 | 4.769 | 20.79 | <0.001 |  |
| Non-Hispanic White | 1.716 | 1.268 | 2.322 | 2.579 | 1.966 | 3.383 | 7.842 | 5.994 | 10.26 | <0.001 |  |
| Non-Hispanic Black | 1.804 | 1.135 | 2.866 | 3.487 | 2.282 | 5.328 | 9.917 | 6.338 | 15.52 | <0.001 |  |
| Other/multiracial | 1.587 | 0.851 | 2.957 | 2.224 | 1.133 | 4.366 | 7.771 | 3.393 | 17.8 | <0.001 |  |
| Education |  |  |  |  |  |  |  |  |  |  | 0.326 |
| High school and below | 1.622 | 1.229 | 2.14 | 2.24 | 1.741 | 2.881 | 7.335 | 5.668 | 9.491 | <0.001 |  |
| Above high school | 1.728 | 1.201 | 2.486 | 3.023 | 2.177 | 4.198 | 8.365 | 6.045 | 11.57 | <0.001 |  |
| Marital |  |  |  |  |  |  |  |  |  |  | 0.512 |
| Having a partner | 1.815 | 1.341 | 2.455 | 3.048 | 2.333 | 3.981 | 8.704 | 6.394 | 11.85 | <0.001 |  |
| Without partner | 1.553 | 1.157 | 2.085 | 2.099 | 1.572 | 2.801 | 7.368 | 5.499 | 9.872 | <0.001 |  |
| PIR |  |  |  |  |  |  |  |  |  |  | 0.879 |
| <1.0 | 1.315 | 0.746 | 2.319 | 2.118 | 1.318 | 3.404 | 5.896 | 3.611 | 9.629 | <0.001 |  |
| 1.0–3.0 | 1.643 | 1.192 | 2.263 | 2.483 | 1.823 | 3.382 | 7.316 | 5.206 | 10.28 | <0.001 |  |
| ≥3.0 | 1.932 | 1.292 | 2.889 | 2.927 | 1.966 | 4.356 | 9.592 | 6.517 | 14.12 | <0.001 |  |
| Smoke |  |  |  |  |  |  |  |  |  |  | 0.293 |
| Never | 1.996 | 1.429 | 2.788 | 2.571 | 1.813 | 3.647 | 9.126 | 6.346 | 13.12 | <0.001 |  |
| Former | 1.922 | 1.239 | 2.98 | 3.373 | 2.201 | 5.169 | 9.258 | 6.098 | 14.06 | <0.001 |  |
| Current | 1.232 | 0.76 | 1.998 | 2.211 | 1.399 | 3.494 | 5.662 | 3.515 | 9.12 | <0.001 |  |

**Abbreviations:** OR, Odds Ratio; PIR, Income -poverty ratio; TyG-BMI Index, triglyceride glucose body mass index; CMM, cardiometabolic multimorbidity

**Supplementary Table 3** Subgroup Analysis of the Association Between METS-IR and CMM.

| **Characteristic** | **33.74-41.03** | | | **41.03-49.9** | | | **≥49.9** | | | **P for trend** | **P for interaction** |
| --- | --- | --- | --- | --- | --- | --- | --- | --- | --- | --- | --- |
|  | **OR** | **lower** | **upper** | **OR** | **lower** | **upper** | **OR** | **lower** | **upper** |  |  |
| Age_group |  |  |  |  |  |  |  |  |  |  | <0.001 |
| <40 | 1.406 | 0.417 | 4.744 | 1.867 | 0.752 | 4.635 | 8.881 | 3.586 | 21.99 | <0.001 |  |
| 40-60 | 1.129 | 0.671 | 1.899 | 2.526 | 1.586 | 4.024 | 7.634 | 4.493 | 12.97 | <0.001 |  |
| ≥60 | 1.777 | 1.39 | 2.271 | 2.954 | 2.221 | 3.928 | 5.256 | 4.048 | 6.826 | <0.001 |  |
| Gender |  |  |  |  |  |  |  |  |  |  | 0.92 |
| female | 1.501 | 1.095 | 2.059 | 2.736 | 1.978 | 3.784 | 6.821 | 4.84 | 9.613 | <0.001 |  |
| male | 1.806 | 1.3 | 2.508 | 3.204 | 2.421 | 4.242 | 9.58 | 6.88 | 13.34 | <0.001 |  |
| Race |  |  |  |  |  |  |  |  |  |  | 0.756 |
| Mexican American | 1.755 | 0.842 | 3.656 | 3.291 | 1.558 | 6.953 | 9.07 | 4.099 | 20.07 | <0.001 |  |
| Other Hispanic | 3.432 | 1.623 | 7.257 | 4.235 | 2.208 | 8.124 | 11.37 | 5.726 | 22.57 | <0.001 |  |
| Non-Hispanic White | 1.547 | 1.111 | 2.155 | 2.921 | 2.179 | 3.914 | 7.972 | 5.792 | 10.97 | <0.001 |  |
| Non-Hispanic Black | 1.975 | 1.183 | 3.297 | 3.8 | 2.373 | 6.085 | 9.771 | 5.867 | 16.27 | <0.001 |  |
| Other/multiracial | 1.562 | 0.881 | 2.768 | 2.066 | 0.953 | 4.479 | 8.385 | 3.494 | 20.12 | <0.001 |  |
| Education |  |  |  |  |  |  |  |  |  |  | 0.669 |
| High school and below | 1.678 | 1.264 | 2.227 | 2.785 | 2.062 | 3.762 | 7.898 | 5.917 | 10.54 | <0.001 |  |
| Above high school | 1.574 | 1.071 | 2.315 | 3.082 | 2.229 | 4.263 | 8.003 | 5.694 | 11.25 | <0.001 |  |
| Marital |  |  |  |  |  |  |  |  |  |  | 0.953 |
| Having a partner | 1.717 | 1.292 | 2.282 | 3.146 | 2.375 | 4.169 | 8.346 | 6.107 | 11.41 | <0.001 |  |
| Without partner | 1.621 | 1.106 | 2.374 | 2.83 | 1.985 | 4.034 | 8.292 | 5.512 | 12.47 | <0.001 |  |
| PIR |  |  |  |  |  |  |  |  |  |  | 0.440 |
| <1.0 | 1.085 | 0.608 | 1.939 | 2.057 | 1.262 | 3.352 | 5.489 | 3.36 | 8.968 | <0.001 |  |
| 1.0–3.0 | 1.573 | 1.114 | 2.222 | 2.642 | 1.92 | 3.635 | 7.24 | 4.821 | 10.87 | <0.001 |  |
| ≥3.0 | 2.121 | 1.411 | 3.189 | 3.975 | 2.633 | 6.002 | 10.74 | 7.627 | 15.13 | <0.001 |  |
| Smoke |  |  |  |  |  |  |  |  |  |  | 0.397 |
| Never | 1.604 | 1.167 | 2.204 | 2.705 | 1.987 | 3.684 | 8.431 | 5.877 | 12.09 | <0.001 |  |
| Former | 2.105 | 1.383 | 3.204 | 3.848 | 2.56 | 5.784 | 9.883 | 6.81 | 14.34 | <0.001 |  |
| Current | 1.377 | 0.826 | 2.296 | 2.844 | 1.766 | 4.58 | 5.944 | 3.58 | 9.869 | <0.001 |  |

**Abbreviations:** OR, Odds Ratio; PIR, Income -poverty ratio; METS-IR, metabolic score for insulin resistance; CMM, cardiometabolic multimorbidity

**Supplementary Table 4** Subgroup Analysis of the Association Between the TG/HDL-C Ratio and CMM.

| **Characteristic** | **1.16-1.92** | | | **1.92-3.64** | | | **≥3.64** | | | **P for trend** | **P for interaction** |
| --- | --- | --- | --- | --- | --- | --- | --- | --- | --- | --- | --- |
|  | **OR** | **lower** | **upper** | **OR** | **lower** | **upper** | **OR** | **lower** | **upper** |  |  |
| Age_group |  |  |  |  |  |  |  |  |  |  | <0.001 |
| <40 | 0.687 | 0.275 | 1.716 | 0.982 | 0.417 | 2.312 | 3.203 | 1.448 | 7.084 | <0.001 |  |
| 40-60 | 1.928 | 1.338 | 2.777 | 2.346 | 1.565 | 3.519 | 4.247 | 2.895 | 6.228 | <0.001 |  |
| ≥60 | 1.581 | 1.245 | 2.009 | 2.523 | 1.949 | 3.264 | 3.991 | 3.11 | 5.123 | <0.001 |  |
| Gender |  |  |  |  |  |  |  |  |  |  | 0.39 |
| female | 1.711 | 1.33 | 2.2 | 2.153 | 1.651 | 2.809 | 4.173 | 3.112 | 5.596 | <0.001 |  |
| male | 1.557 | 1.173 | 2.067 | 2.607 | 1.944 | 3.496 | 4.358 | 3.154 | 6.024 | <0.001 |  |
| Race |  |  |  |  |  |  |  |  |  |  | 0.865 |
| Mexican American | 1.435 | 0.809 | 2.543 | 1.91 | 1.047 | 3.487 | 3.194 | 1.757 | 5.806 | <0.001 |  |
| Other Hispanic | 1.639 | 0.846 | 3.174 | 2.338 | 1.197 | 4.564 | 4.096 | 2.18 | 7.695 | <0.001 |  |
| Non-Hispanic White | 1.598 | 1.237 | 2.065 | 2.399 | 1.775 | 3.242 | 4.533 | 3.428 | 5.993 | <0.001 |  |
| Non-Hispanic Black | 2.056 | 1.493 | 2.83 | 2.484 | 1.752 | 3.521 | 4.332 | 2.89 | 6.493 | <0.001 |  |
| Other/multiracial | 1.004 | 0.51 | 1.978 | 2.008 | 0.977 | 4.127 | 3.496 | 1.519 | 8.048 | 0.001 |  |
| Education |  |  |  |  |  |  |  |  |  |  | 0.217 |
| High school and below | 1.558 | 1.15 | 2.112 | 1.861 | 1.413 | 2.452 | 3.738 | 2.829 | 4.94 | <0.001 |  |
| Above high school | 1.633 | 1.192 | 2.237 | 2.791 | 2.039 | 3.82 | 4.617 | 3.483 | 6.12 | <0.001 |  |
| Marital |  |  |  |  |  |  |  |  |  |  | 0.040 |
| Having a partner | 2.016 | 1.561 | 2.604 | 3.057 | 2.334 | 4.004 | 4.995 | 3.75 | 6.654 | <0.001 |  |
| Without partner | 1.21 | 0.877 | 1.668 | 1.581 | 1.168 | 2.142 | 3.705 | 2.658 | 5.166 | <0.001 |  |
| PIR |  |  |  |  |  |  |  |  |  |  | 0.488 |
| <1.0 | 1.369 | 0.869 | 2.154 | 1.768 | 1.117 | 2.797 | 3.088 | 1.987 | 4.8 | <0.001 |  |
| 1.0–3.0 | 1.349 | 1.025 | 1.775 | 1.876 | 1.418 | 2.483 | 3.361 | 2.458 | 4.594 | <0.001 |  |
| ≥3.0 | 2.176 | 1.546 | 3.062 | 3.422 | 2.383 | 4.913 | 6.177 | 4.279 | 8.918 | <0.001 |  |
| Smoke |  |  |  |  |  |  |  |  |  |  | 0.701 |
| Never | 1.728 | 1.286 | 2.322 | 2.277 | 1.697 | 3.055 | 4.252 | 3.086 | 5.859 | <0.001 |  |
| Former | 1.436 | 0.999 | 2.064 | 2.514 | 1.734 | 3.645 | 4.762 | 3.33 | 6.809 | <0.001 |  |
| Current | 1.453 | 0.838 | 2.519 | 1.832 | 1.107 | 3.033 | 3.007 | 1.895 | 4.772 | <0.001 |  |

**Abbreviations:** OR, Odds Ratio; PIR, Income -poverty ratio; TG/HDL-C Ratio, triglyceride to high-density lipoprotein cholesterol ratio; CMM, cardiometabolic multimorbidity

**Supplementary Table 5** Subgroup Analysis of the Association Between HOMA-IR and CMM.

| **Characteristic** | **1.34-2.33** | | | **2.33-4.06** | | | **≥4.06** | | | **P for trend** | **P for interaction** |
| --- | --- | --- | --- | --- | --- | --- | --- | --- | --- | --- | --- |
|  | **OR** | **lower** | **upper** | **OR** | **lower** | **upper** | **OR** | **lower** | **upper** |  |  |
| Age_group |  |  |  |  |  |  |  |  |  |  | <0.001 |
| <40 | 0.451 | 0.189 | 1.074 | 0.68 | 0.333 | 1.388 | 3.921 | 1.807 | 8.507 | <0.001 |  |
| 40-60 | 1.08 | 0.643 | 1.813 | 2.647 | 1.715 | 4.085 | 9.951 | 6.708 | 14.76 | <0.001 |  |
| ≥60 | 1.3 | 0.986 | 1.715 | 2.204 | 1.693 | 2.87 | 4.781 | 3.584 | 6.378 | <0.001 |  |
| Gender |  |  |  |  |  |  |  |  |  |  | 0.13 |
| female | 1.347 | 0.969 | 1.871 | 2.845 | 2.111 | 3.834 | 7.366 | 5.374 | 10.1 | <0.001 |  |
| male | 1.027 | 0.727 | 1.45 | 1.746 | 1.272 | 2.396 | 6.264 | 4.509 | 8.702 | <0.001 |  |
| Race |  |  |  |  |  |  |  |  |  |  | 0.561 |
| Mexican American | 1.464 | 0.681 | 3.149 | 2.284 | 1.194 | 4.37 | 6.07 | 3.057 | 12.06 | <0.001 |  |
| Other Hispanic | 1.08 | 0.449 | 2.596 | 2.119 | 1.01 | 4.446 | 8.944 | 4.066 | 19.68 | <0.001 |  |
| Non-Hispanic White | 1.129 | 0.818 | 1.559 | 2.263 | 1.67 | 3.066 | 7.175 | 5.215 | 9.872 | <0.001 |  |
| Non-Hispanic Black | 1.084 | 0.69 | 1.702 | 1.771 | 1.159 | 2.706 | 5.669 | 3.727 | 8.622 | <0.001 |  |
| Other/multiracial | 1.212 | 0.549 | 2.68 | 2.496 | 1.225 | 5.084 | 5.199 | 2.362 | 11.44 | <0.001 |  |
| Education |  |  |  |  |  |  |  |  |  |  | 0.092 |
| High school and below | 1.109 | 0.773 | 1.592 | 1.95 | 1.419 | 2.679 | 5.59 | 4.012 | 7.791 | <0.001 |  |
| Above high school | 1.149 | 0.786 | 1.679 | 2.416 | 1.689 | 3.456 | 8.085 | 5.803 | 11.26 | <0.001 |  |
| Marital |  |  |  |  |  |  |  |  |  |  | 0.009 |
| Having a partner | 0.969 | 0.698 | 1.346 | 2.432 | 1.769 | 3.344 | 6.49 | 4.617 | 9.123 | <0.001 |  |
| Without partner | 1.621 | 1.143 | 2.299 | 1.927 | 1.394 | 2.663 | 7.949 | 5.8 | 10.89 | <0.001 |  |
| PIR |  |  |  |  |  |  |  |  |  |  | 0.006 |
| <1.0 | 1.614 | 0.935 | 2.787 | 2.183 | 1.371 | 3.477 | 5.211 | 3.279 | 8.28 | <0.001 |  |
| 1.0–3.0 | 0.853 | 0.625 | 1.164 | 1.999 | 1.527 | 2.616 | 5.229 | 3.876 | 7.054 | <0.001 |  |
| ≥3.0 | 1.383 | 0.913 | 2.094 | 2.447 | 1.612 | 3.714 | 9.943 | 6.688 | 14.78 | <0.001 |  |
| Smoke |  |  |  |  |  |  |  |  |  |  | 0.182 |
| Never | 1.145 | 0.797 | 1.647 | 2.173 | 1.525 | 3.097 | 6.782 | 4.806 | 9.571 | <0.001 |  |
| Former | 1.527 | 1.033 | 2.256 | 2.648 | 1.833 | 3.827 | 8.889 | 5.97 | 13.24 | <0.001 |  |
| Current | 0.848 | 0.532 | 1.351 | 2.166 | 1.465 | 3.201 | 4.877 | 3.208 | 7.414 | <0.001 |  |

**Abbreviations:** OR, Odds Ratio; PIR, Income -poverty ratio; HOMA-IR, homeostasis model assessment of insulin resistance; CMM, cardiometabolic multimorbidity
